# Editorial: The immune system and inflammation in musculoskeletal health, aging, and disease

**DOI:** 10.3389/fimmu.2023.1218118

**Published:** 2023-05-18

**Authors:** Gurpreet S. Baht, Matthew W. Grol

**Affiliations:** ^1^ Department of Orthopaedic Surgery, Duke Molecular Physiology Institute, Duke University, Durham, NC, United States; ^2^ Department of Physiology and Pharmacology, Schulich School of Medicine & Dentistry, University of Western Ontario, London, ON, Canada

**Keywords:** inflammation, immune system, musculoskeletal disorders, musculoskeletal system, osteoarthritis, rheumatoid arthritis, intervertebral disc degeneration, dental implants

The musculoskeletal and immune systems are intricately linked in anatomical space and function, with crosstalk between immune cells and musculoskeletal tissues, including bone, cartilage, muscle, and tendons, being essential for normal development and homeostasis (1–4). Such a relationship is also critical during injury and repair, with inflammation and immune cells being necessary for initiating and resolving injury-induced tissue responses and altered extracellular matrix composition and turnover, likewise regulating immune cell engagement (5–9). Over the last decade, growing evidence has demonstrated that alterations to immune cell populations caused by aging and metabolic dysfunction underly the impaired tissue repair responses seen in chronic musculoskeletal diseases and acute injuries, including those affecting synovial joints (e.g., osteoarthritis), bones (e.g., osteoporosis, fracture healing), muscles (e.g., sarcopenia), and tendons/ligaments (e.g., tendinopathy, rupture).

The burden from musculoskeletal conditions continues to rise globally, impacting patients’ quality of life, independence, and health, social and economic systems due to increasing care costs and work loss. Over 1.7 billion people globally live with musculoskeletal conditions, according to the World Health Organization (10) and findings from the Lancet’s Global Burden of Disease Study 2019 (11–13). Low back pain is the main contributor to this overall burden, while osteoarthritis (OA) shows the most rapid increase of these conditions. While advances have been made in treating osteoporosis in the past decade, intervertebral disc degeneration (IVDD), OA, and many others where disease pathogenesis is less understood lack disease-modifying therapeutics. Progress in understanding how diseases such as IVDD and OA progress has revealed an essential role for inflammatory dysregulation in these conditions; however, several important questions must be resolved before this can be mobilized as a therapeutic strategy. In this Frontiers of Immunology – Inflammation Section Research Topic; The Immune System and Inflammation in Musculoskeletal Health, Aging, and Disease*;* we present a collection of articles focused on the aging skeleton and aim to shed light on age-associated dysregulation of inflammatory processes that lead to musculoskeletal morbidity with the hope of identifying targets for future disease-modifying therapeutics (**Figure 1**).

**Figure 1:**
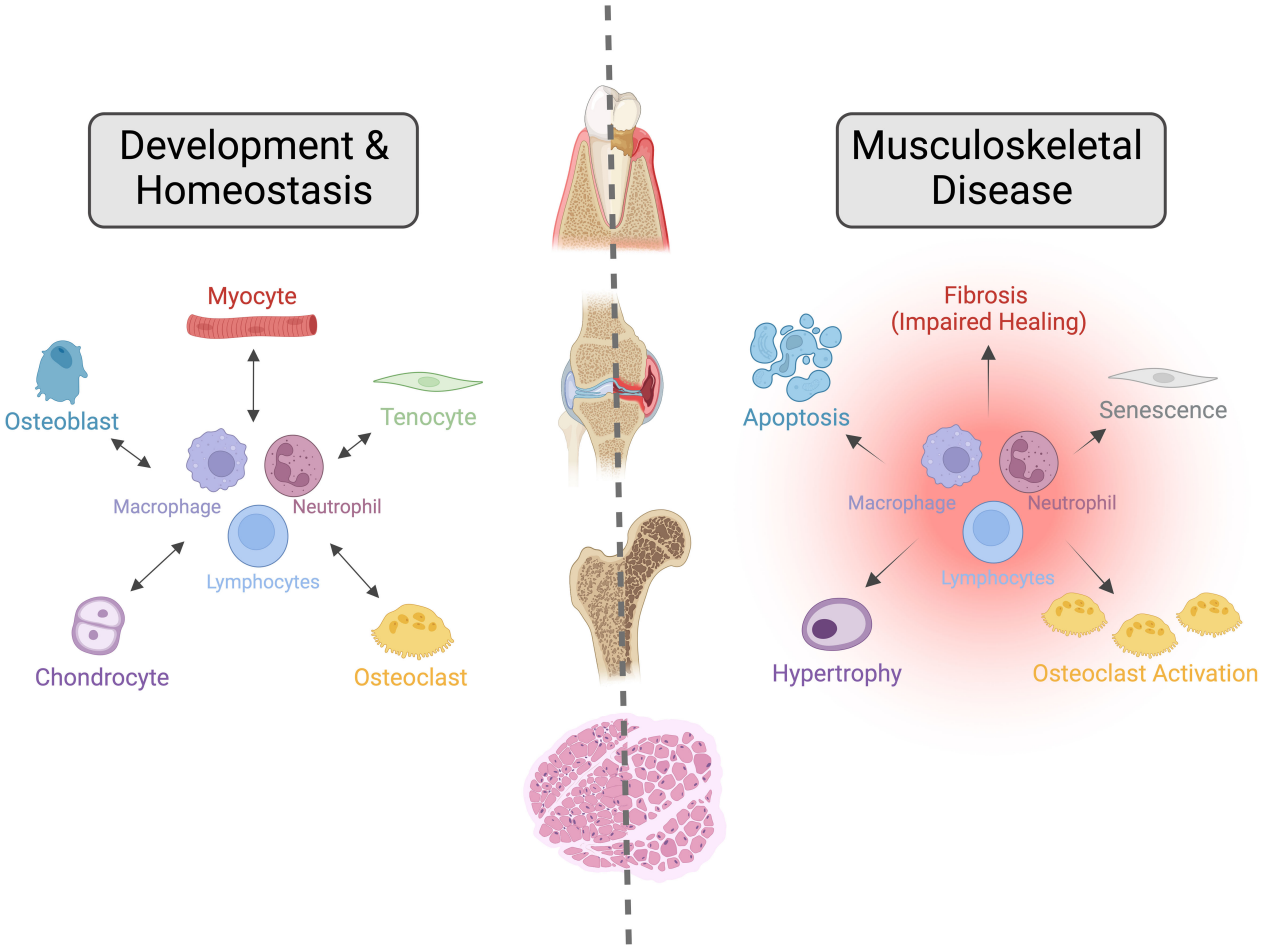
The Importance of Crosstalk between the Immune and Musculoskeletal Systems during Development, Homeostasis, and Disease. Interactions between the immune and musculoskeletal systems are critical for their development and homeostasis. Alterations to these interactions caused by aging, metabolic dysfunction, or genetic mutations lead to impaired repair responses and dysregulated tissue homeostasis, resulting in various musculoskeletal diseases such as osteoarthritis, osteoporosis, sarcopenia, periodontal disease and dental implant loosening, and intervertebral disc degeneration. In this Frontiers of Immunology – Inflammation Section research topic, we present a collection of articles that aim to shed light on age-associated dysregulation of inflammatory processes that lead to musculoskeletal morbidity. Image created with BioRender.com under Academic License.

OA is a multifactorial disease affecting synovial joints and one of the most common musculoskeletal diseases worldwide (14, 15). The disease is characterized by progressive loss of articular cartilage, subchondral bone and peri-articular bone remodeling, and intra-articular inflammation with synovitis, culminating in chronic pain and reduced mobility. The role of immune cells in OA progression was long recognized but remains poorly understood. In this Research Topic, Haubruck et al. outline the role of monocytic cells in post-traumatic OA (PTOA) and discuss therapeutic interventions to potentially improve disease outcomes. In addition to modifying disease outcomes, improved metrics for classifying OA severity are required to better manage the disease and for the development of disease-modifying drugs. In this regard, the search for biomarkers that can identify the stage of OA progression and distinguish between OA at different anatomical sites has become increasingly important. In this Research Topic, Zhang et al. identify extracellular vesicle sub-species that are more prevalent in patients with knee OA and carry a pro-inflammatory cargo, including tumor necrosis factor-alpha (TNF-α). In a second study in this Research Topic, Ratneswaran et al. investigated patients with hand OA and identified that circulating cytokines could distinguish OA severity of the trapeziometacarpal joint. Specifically, interleukin-7 (IL-7) was identified as a marker capable of differentiating disease severity with higher levels associated with a decreased likelihood of trapeziometacarpel joint OA needing surgical intervention. In terms of targeting inflammation to treat OA, Vachhani et al. investigated whether CD200R1 agonists could delay the progression of PTOA; however, neither the protein therapeutic CD200Fc nor the synthetic DNA aptamer CCS13 were able to attenuate cartilage degeneration or synovitis, despite their ability to blunt inflammatory response in the knee. This study points to the complexities of targeting inflammation in complex diseases such as OA.

In recent years, synoviocytes and resident immune cells within the synovium have emerged as key players in the progression of OA and other joint diseases (16, 17). In this Research Topic, Jones et al. used ChIP-seq to assay fibroblast-like synoviocytes and identified that SOX4 and RELA physically interact on chromatin, with TNF-responsive genes being the primary targets of this transcriptional complex. Sodhi et al. identified sex-dependent differences between complement and synovial microvascular pathology in patients, with higher synovial fluid C5 levels being associated with increased complement activation and decreased synovial vascularization in males but not in females with OA. Farina et al. identified that the binding of pro-nerve growth factor to p75NTR on synoviocytes elicits an inflammatory response, resulting in the release of IL-1β, IL-6 and TNF-α. Inhibition of this binding prevented this inflammatory response and represents a novel therapeutic approach in chronic arthritis. Finally, the role of Hippo pathway targets, the mechanoresponsive Yap/Taz transcription factors, was investigated in the context of rheumatoid arthritis (RA) by Caire et al., where the authors demonstrated that RA activates Yap/Taz within synoviocytes. Treatment with a Yap/Taz inhibitor reversed the RA phenotype indicating their transcriptional inhibition could be relevant to treat inflammatory-related diseases.

Aging also affects other tissues of the skeleton. The effect of oxidative stress on IVDD was studied by Cao et al. Specifically, this group found that *Ccnb1* and *Pkd1* help to regulate oxidative stress during intervertebral disc degeneration and lead to CD8+ T cell infiltration. This work presents *Ccnb1* & *Pkd1* as potential targets for treatment in IVDD. A lesser-considered modality of the aging skeleton is dental health. Schluessel et al. investigated the loosening of dental implants and related gene expression profiles with the loosening of orthopedic implants. Using co-culture systems, disparate and overlapping gene profiles were established, identifying potential therapeutic targets for improving and maintaining the integration of dental and orthopedic implants. Ma et al. investigated the ratio of pro-inflammatory and anti-inflammatory macrophages within the infrapatellar fat pad and subcutaneous fat tissue of patients receiving total knee arthroplasties. The macrophage ratio differed between infrapatellar and subcutaneous fat, with the infrapatellar environment presenting a more inflammatory niche that could be targeted for therapeutic intervention in joint disease.

The works within this collection shed light on the importance of immune cell populations and signaling mechanisms in musculoskeletal disease progression; however, more work is certainly needed before therapeutics targeting these mechanisms can be developed to modify complex diseases such as OA and IVDD.

In conclusion, we thank the contributing authors for sharing their findings and insights in these critical areas. Musculoskeletal diseases have long presented a challenge to the medical and scientific communities. However, with an improved understanding of how the immune and musculoskeletal systems interact, better and more selective therapies could be on the horizon.

## Author contributions

All authors listed have made equal substantial, direct, and intellectual contributions to the work and approved it for publication.
